# Exploring the Connections Between Grip Strength, Nutritional Status, Frailty, Depression, and Cognition as Initial Assessment Tools in Geriatric Rehabilitation—A Pilot Study

**DOI:** 10.3390/medicina60121916

**Published:** 2024-11-21

**Authors:** Amalia Teodora Vancea Nemirschi, Andreea Alexandra Lupu, Kamer-Ainur Aivaz, Mădălina Gabriela Iliescu, Michel Deriaz, Mircea Marzan, Luiza Spiru

**Affiliations:** 1 Faculty of Medicine, Ovidius University of Constanta, University Alley No. 1, 900470 Constanta, Romania; amalia.vancea@365.univ-ovidius.ro (A.T.V.N.); aa.lupu@365.univ-ovidius.ro (A.A.L.); 2 County Clinical Emergency Hospital of Constanta “Sf Apostol Andrei”, Boulevard Tomis No. 145, 900591 Constanta, Romania; 3 Doctoral School, University of Medicine and Pharmacy Carol Davila, Dionisie Lupu Street, No. 37, Sector 2, 020021 Bucharest, Romaniamircea.marzan@drd.umfcd.ro (M.M.); 4 Faculty of Economic Science, Ovidius University of Constanta, University Alley No. 1, 900470 Constanta, Romania; kamer.aivaz@365.univ-ovidius.ro; 5 Ana Aslan International Foundation, The Excellence Memory Centre, Brain Health and Longevity Science, Spatarului Street No. 3, Sector 2, 030167 Bucharest, Romania; 6 Genève College of Longevity Science, Rue de la Corraterie 5, 1204 Genève, Switzerland; 7 University of Applied Sciences of Western Switzerland, Rue de la Tambourine 17, 1227 Carouge, Switzerland

**Keywords:** grip strength, nutritional status, frailty, depression, cognition, geriatric rehabilitation, geriatric assessment, digital dynamometer

## Abstract

*Background and Objective:* In the context of the rapidly aging global population, the older adult vulnerability poses a significant challenge for public health systems. Frailty, cognitive and nutritional status, depression, and grip strength are essential parameters for staging the vulnerability of older adults. The objective of this study is to identify a rapid but multidimensional geriatric assessment tool that can enhance the rehabilitation process for older adults, tailored to their specific needs. *Materials and Methods:* This pilot study examines the relationships between grip strength, nutritional status, frailty, depression, and cognition in a group of 80 older adults with a mean age of 69.6 years, 49 male and 31 female, using standardized geriatric scales and digital grip strength measurements. The study employed a digital dynamometer, a portable and reliable tool that facilitated quick and accurate grip strength measurements. *Results:* The analysis revealed significant correlations among the parameters. Greater grip strength was associated with better cognitive performance (r = 0.237, *p* = 0.034) and improved nutritional status (r = 0.267, *p* = 0.016), while it was inversely related to frailty (r = −0.313, *p* = 0.005). Nutritional status also played a key role, showing an inverse relationship with frailty (r = −0.333, *p* = 0.003) and depression levels (r = −0.248, *p* = 0.027). Furthermore, frailty and depression were strongly interconnected, with those experiencing higher frailty levels also displaying more severe depressive symptoms (r = 0.545, *p* < 0.001). Marital status was also relevant: married participants exhibited higher grip strength, lower frailty, and fewer depressive symptoms, suggesting that social support positively influences both physical and mental health in older adults. *Conclusions:* These findings not only emphasize the need for integrated care approaches that simultaneously address physical health, nutrition, and cognitive function, but also provide a foundation for the development of a rapid and multidimensional assessment protocol, which consists of using a digital dynamometer and four geriatric scales. Such a tool could play a crucial role in the early detection of frailty syndrome and guide the implementation of multidisciplinary, tailored therapeutic strategies aimed at preserving the autonomy and improving the quality of life of older adults.

## 1. Introduction

In the context of the global demographic shift characterized by an aging population, the increased vulnerability of older adults presents a pressing challenge for public health systems and the global economy. Frailty syndrome, a state marked by heightened susceptibility to external stressors, is a well-recognized indicator of this vulnerability and is associated with increased dependence, hospitalization rates, and a higher demand for medical resources, all of which impose a growing economic burden on societies [1]. This underscores the urgent need for effective strategies in the early detection and prevention of frailty, which can help older adults maintain their independence and quality of life for longer [2].

The vulnerability of elderly patients is often defined in terms of physical frailty, cognitive status, nutritional status, depression, and grip strength—all essential markers for staging frailty syndrome. Frailty syndrome is understood as a complex, multidimensional state of decreased physiological reserves, affecting multiple body systems and leading to a greater susceptibility to adverse outcomes, and even premature mortality [3,4,5]. Preventive strategies are, therefore, essential to mitigate these risks and improve health outcomes in this vulnerable population [6].

To facilitate early diagnosis, the comprehensive geriatric assessment (CGA) has become a foundational tool in clinical practice, offering a holistic view of an older adult’s health status through integrated physical, cognitive, and emotional assessments [7]. Common tools used in the CGA, such as the Yesavage Geriatric Depression Scale [8], the Rapid Cognitive Test [9], the Edmonton Frailty Scale [10], and the Mini Nutritional Assessment (MNA) [11], are well established in geriatric care for assessing depressive symptoms, cognitive decline, and frailty, thus guiding the development of personalized interventions. However, a complex CGA usually uses more than these four evaluation scales, which makes it time-intensive and requires specialized infrastructure, which may limit its feasibility in everyday practice across various specialties. Given the increasing population of older adults, there is a need for a simpler, more rapid assessment instrument that remains clinically comprehensive yet efficient in terms of time and cost.

Among the critical components of the CGA is grip strength measurement, a reliable biomarker of neuromuscular health and physical function that has been closely linked to cognitive function and physical frailty. Research has shown that lower grip strength is associated with higher risks of cognitive decline, dementia [12,13,14,15], and depression, highlighting the importance of incorporating grip strength into routine geriatric evaluations as an early marker for both physical and psychological vulnerabilities [16].

To make this assessment process more accessible and efficient, the use of innovative tools such as the Squegg™ Smart Dynamometer can significantly enhance clinical practice [17].

**Figure 1:**
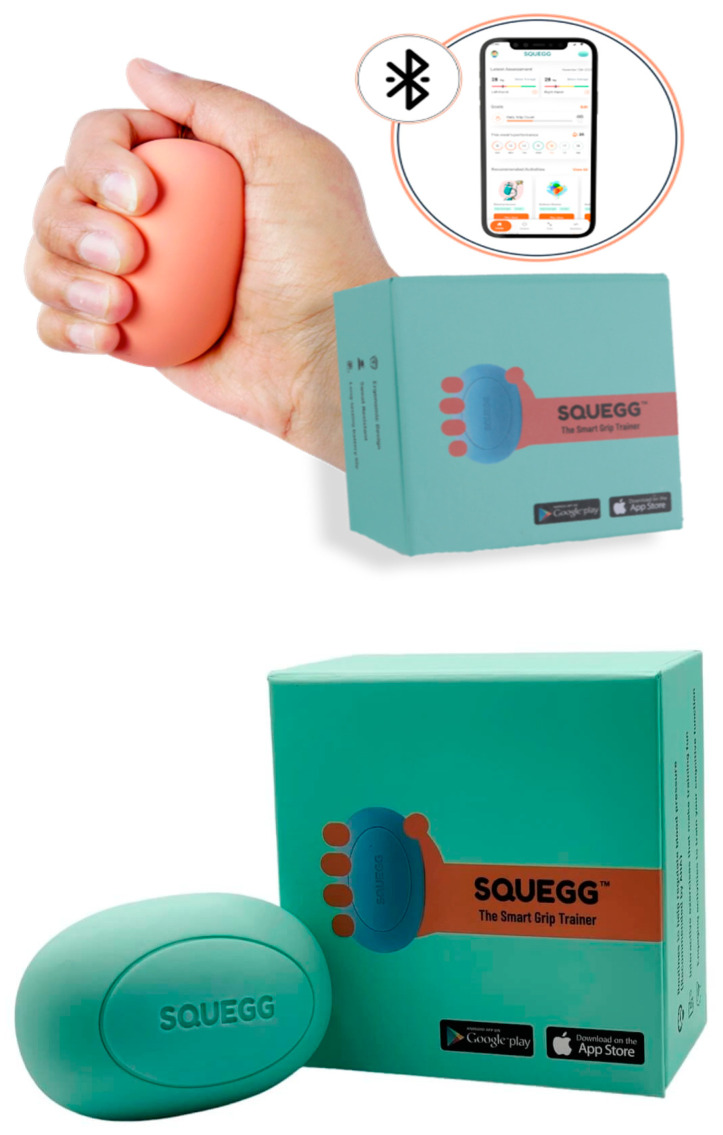
Squegg™ Smart Dynamometer, source: https://www.mysquegg.com/products/squegg-digital-grip-strengthener (accessed on 14 October 2024).

The Squegg™ dynamometer (Figure 1) offers several advantages that makes it highly feasible for use in a busy clinical setting. Unlike traditional devices, Squegg™ is lightweight, portable, and easy to operate, featuring an ergonomic design that increases patient comfort. Its Bluetooth-enabled functionality allows real-time data collection and biofeedback, facilitating faster and more precise assessments of grip strength. These characteristics make it particularly suitable for a rapid, comprehensive geriatric assessment, ensuring that grip strength measurements can be integrated seamlessly into regular evaluations without adding significant time or complexity to the process [18]. The practical nature of the Squegg™ dynamometer makes it an ideal tool not only for geriatric specialists but also for other healthcare professionals who work with older adults, such as general practitioners, nurses, and rehabilitation therapists [19,20,21]. 

This pilot study explores the interrelationships between grip strength, nutritional status, frailty, depression, and cognition in a cohort of older adults, with the goal of evaluating whether a brief yet multidimensional geriatric assessment—combining four geriatric scales and the Squegg™ Smart Dynamometer—can serve as a practical and implementable tool for healthcare professionals caring for adults over 65. Our approach responds to the need for a rapid and reliable assessment by integrating these widely used scales with the Squegg™ dynamometer, a portable and user-friendly digital device for measuring grip strength. This simplified tool enables specialists across various fields, particularly in rehabilitation, to adopt a multidisciplinary approach to assessing physical, cognitive, and emotional health. By confirming correlations among grip strength, cognitive status, nutritional status, depression, and frailty, we aimed to validate our hypothesis that this streamlined instrument aligns with findings in the current literature, supporting its potential as a practical tool for early frailty detection and management.

Our study formulates the following hypotheses:

**Hypothesis 1.**  *There is a significant correlation between grip strength and cognitive function, suggesting that reduced grip strength is associated with cognitive decline.*

**Hypothesis 2.**  *Nutritional status, measured by the Mini Nutritional Assessment (MNA), is inversely correlated with frailty and depression, indicating that better nutritional status may mitigate frailty and cognitive decline.*

**Hypothesis 3.**  *Marital status affects overall health, with unmarried older adults exhibiting higher frailty and depression levels than their married counterparts.*

**Hypothesis 4.**  *This multidimensional, easy to use geriatric assessment instrument aligns with findings in the current literature, supporting its potential as a practical tool for early frailty detection and management in geriatric rehabilitation.*

## 2. Materials and Methods

A cross-sectional study design was used to measure the variables at a specific point in time, providing a snapshot of participants’ health status. The questionnaire and muscle strength measurement were applied to patients who presented in the outpatient department of Physical Medicine and Rehabilitation of the County Clinical Emergency Hospital of Constanta—St. Apostle Andrew, between January and April 2024. Data were collected and recorded by attending physicians using standardized methods to ensure the consistency and reliability of measurements.

### 2.1. Sample Description

The questionnaire included questions about patients’ personal data (name, surname, age, occupation, marital status), principal and secondary diagnoses, personal pathologic and inherited history, height, weight, and the described rating scales: Edmonton Frailty Scale, Mini Nutritional Assessment (MNA), Yesavage Geriatric Depression Scale (GDS), and the Rapid Cognitive Test (RCT).

Inclusion criteria. Patients who presented with a principal diagnosis of vertebral-peripheral arthritic disease. Females and males over 65 years of age.

Exclusion criteria. Patients with neurologic pathology, diabetic neuropathy, acute trauma, or post-traumatic sequelae in the upper limbs, arthritis in upper limb.

The study is based on a sample of 80 people over the age of 65. Although the sample size may seem modest, it is important to analyse the specific context of the study to ensure its representativeness in order to obtain reliable results. In general, in studies dealing with complex phenomena and multiple variables, such as those related to the health of older adults, a sample of this size is sufficient to detect significant effects or trends in the data, provided that the sample distribution reflects the structure of the general population in terms of age, gender, health status, and other relevant variables.

From a statistical point of view, the choice of the sample took into account four key elements: effect size, statistical power, level of significance (alpha), and variation in the data. To ensure a statistical power of 80% (1-β = 0.80) to detect a significant difference, with a significance level of 0.05 (α = 0.05) and an estimated effect size of 0.5 (according to Cohen’s criteria for medium effect sizes), G*Power analysis indicates a minimum required sample size of 54 participants for a t-test (difference between two dependent means-matched pairs) [22].

In this study, the gender and age distributions show a diversity that gives us a fair basis for generalization. Furthermore, the representativeness of the sample is closely related to the quality and accuracy of the data collected.

### 2.2. Instruments

Grip strength was quantified using the Squegg™ digital dynamometer, a validated tool known for its portability, accuracy, and real-time feedback, making it ideal for clinical use in both routine assessments and rehabilitation settings. The Squegg™ dynamometer combines ergonomic design and Bluetooth-enabled functionality, facilitating efficient grip strength measurement with immediate data feedback. This makes it well-suited for older adults, enhancing patient comfort and allowing for precise longitudinal analysis. Ciro et al. (2022) demonstrated its efficiency in tracking muscle strength recovery in adults recovering from COVID-19 [21], while Varadarajan et al. (2024) showed its utility in identifying adults at nutritional risk, emphasizing its ease of use for healthcare professionals [19]. Additionally, Stamate et al. (2023) highlighted the broader clinical applicability of digital dynamometers like Squegg™, noting their established role in assessing the risk of morbidity and mortality through grip strength measurement. Compared to traditional dynamometers like the Jamar^®^ Hydraulic Hand Dynamometer, Squegg™ offers improved portability and comfort, making it a reliable option for both routine clinical practice and specialized rehabilitation programs [17,18,20].

Nutritional status is assessed by the Mini Nutritional Assessment (MNA), which includes questions about dietary history, assessment of weight loss, and measurement of anthropometric indices. This tool provides a comprehensive overview of the risk of malnutrition. Mini Nutritional Assessment (MNA): This scale is essential for assessing the nutritional status of older people. The MNA includes 17 questions on food intake, recent weight loss, mobility, and other indicators of malnutrition, with a scoring system that categorizes results as “malnourished” (less than 17 points), “at risk of malnutrition” (17–23.5 points), or “normal nutritional status” (more than 23.5 points).

The Edmonton Frailty Scale (EFS) is a multidimensional assessment tool that quantifies the presence and severity of frailty in older adults. This instrument is employed for the purpose of determining the degree of frailty in older adults. The EFS assesses nine domains, including general health, social and cognitive functioning, and provides a score that reflects the severity of frailty. The final scoring categorizes the results as follows: “not frail” (0–5 points)”, vulnerable” (6–7 points), “mild frailty” (8–9 points)”, moderate frailty” (10–11 points)”, and severe frailty” (12–17 points).

Depression is assessed using the Short Yesavage Geriatric Depression Scale (GDS), an established method that identifies depressive symptoms by means of specific questions designed to be readily comprehensible to older adults. The Yesavage Geriatric Depression Scale is employed for the purpose of evaluating the presence of depressive symptoms in older individuals. The scale comprises 15 yes/no questions pertaining to the patient’s affective experience (mood, interest in activities, energy levels, social engagement, cognitive focus, sleep patterns, general satisfaction). The final scoring categorizes the results as follows: “normal” (0–4 points), “mild depression” (5–8 points), “moderate depression” (9–11 points)”, and severe depression” (12–15 points).

Cognitive function is assessed by the Rapid Cognitive Test (RCT), which is designed to quickly detect signs of cognitive decline through tests of memory and attention. The Rapid Cognitive Test (CRT) is used for rapid screening of cognitive impairment, including dementia and mild cognitive decline. It includes 4 items that cover orientation, recall, and visuospatial abilities with a score range from 0 to 10 that categorizes results as follows: “normal cognitive function” (8–10 points), “mild cognitive impairment” (6–7 points), and “possible dementia or significant cognitive impairment” (0–5 points).

These standardized instruments are well-established for their sensitivity and specificity in geriatric populations, allowing for a comprehensive evaluation of physical, mental, and nutritional health.

### 2.3. Data Collecting Procedures

Each participant was assessed by the treating physician using the same standardized methods to ensure the consistency and reliability of measurement, key elements in scientific studies.

Handgrip strength was measured for each participant using the Squegg™ digital dynamometer, with three trials conducted alternately on both hands. The device provided grip force measurements in kilograms for each hand, and normative values on the Squegg™ platform, adjusted for age and sex, were used to classify participants’ grip strength as normal or weak.

Prior to the measurements, each participant received detailed instructions on how to properly use the device, including when to squeeze, how long to maintain the grip, and when to switch hands. These instructions ensured consistency and accuracy in the measurements. Participants were instructed to hold the device in their palm, with four fingers resting on the ridges and the thumb gripping it securely. The measurement hand was kept unsupported, with the elbow flexed at a 90-degree angle.

The Squegg™ was synchronized with its app via Bluetooth, which recorded all measurements in kilograms and self-zeroed before each use. The three scores for each hand were averaged to determine the final grip strength score for each hand.

In addition to handgrip measurements, we applied the Edmonton Frailty Scale, Yessavage Geriatric Depression Scale, Mini Nutritional Assessment (MNA), and Rapid Cognitive Screen. These assessments, alongside demographic and health information—including age, sex, previous occupation, education level, and personal medical history—provided a complex evaluation of each participant’s functional, psychological, and cognitive status. The entire protocol, encompassing the handgrip measurement and geriatric scales, was completed in approximately 10–15 min per participant.

This multidimensional protocol was developed to provide robust data for statistical correlations between handgrip strength and participants’ psychophysical and cognitive status. By integrating handgrip measurements with geriatric assessments, this approach validates the protocol’s efficiency and reliability in evaluating health and functionality within a geriatric population.

### 2.4. Data Analysis

For data analysis, SPSS version 28 software was used, applying several statistical tests to assess relationships and differences between variables. Statistical description was carried out using means and standard deviations, and data distribution was analysed using skewness and kurtosis coefficients. Normality of distribution was tested using the Shapiro–Wilk test.

Pearson (for normally distributed variable) and Spearman’s (for non-normally distributed variables) correlations were computed to assess the linear relationship between sex, age, marital status, education level, and MNA GDS, RCT, HGS, and EFS scores. Separate Independent samples T tests (for normally distributed variables) and Mann–Whitney U tests (for non-normally distributed variables) were run to identify differences group (sex or marital status) differences. Chi-square test was used to investigate sex differences in the level of education (high school education vs. graduate education). Simple regression analyses were run to investigate whether nutritional status can predict cognitive status (RCT), frailty (EFS), and depression (we used log transformed data for depression).

## 3. Results

Demographic data are presented in Table 1. The means and standard deviations for grip strength (HGS), nutritional status (MNA), frailty (EFS), depression (GDS), and cognition (RCT) in scores for men and women are presented in Table 2. Skewness, kurtosis, and normality tests for each variable are presented in Table 3. Scores, as assessed by the Shapiro–Wilk’s test, were normally distributed for MNA (*p* = 0.110) and HGS (*p* = 0.313), but not for GDS (*p* < 0.001), EFS (*p* = 0.002) and RCT (*p* < 0.001).

### 3.1. Correlations in the Entire Population

Hand grip strength (HGS) showed the greatest correlation with sex (*r*(80) = −0.512 *p* < 0.001), higher HGS, and being associated with being married (*r*(80) = −0.404 *p* < 0.001). We also found positive correlations between HGS and cognitive status (RCT) *r*(80) = 0.237, *p* = 0.034, and between HGS and nutritional status, *r*(80) = 0.267, *p* = 0.016, and negative associations between hand grip strength (HGS) and frailty (EFS) *r*(80) = −0.313, *p* = 0.005. Better nutritional status (MNA) was also associated with lower frailty (EFS) *r*(80) = −0.333, *p* = 0.003, and with lower levels of depression (GDS) *r*(80) = −0.248, *p* = 0.027. There was also a strong positive association between frailty (EFS) and depression (GDS) r(80) = 0.545, *p* < 0.001. Being married was associated with higher hand grip strength HGS r(80) = 0.404, *p* < 0.001 and with lower levels of depression (GSD) and frailty (EFS). Higher age was associated with higher depression GDS *r*(80) = 0.221, *p* = 0.048, and higher fragility (EFS) *r*(80) = 0.244, *p* = 0.030.

### 3.2. Correlations in Men and Women Groups

In the women group, hand grip strength (HGS) was positively associated with nutritional status (MNA) *r*(49) = 0.384, *p* = 0.014, being married (*r*(49) = 0.293, *p* = 0.041, and cognitive status (RCT) *r*(49) = 0.405, *p* = 0.004, and negatively associated with frailty (EFS) *r*(49) = −0.377, *p* = 0.008 and depression (GDS) *r*(49) = −0.356, *p* = 0.012. In the men group, hand grip strength (HGS) was positively associated with being married (*r*(31) = 0.445, *p* = 0.012 and negatively associated with age (*r*(31) = 0.399, *p* = 0.026.

### 3.3. Sex Differences

Hand grip strength was significantly higher [r *t*(78) = 5.70, *p* <.001] for men (M = 30.82, SD = 7.33) compared to women (M = 22.70, SD = 6.30). No statistically significant sex differences were found for MNA, GDS, EFS, RCT, or for levels of education.

### 3.4. Nutritional Status

Simple linear regression analysis was conducted to evaluate the extent to which nutritional status (MNA) could predict cognitive status (RCT), frailty (EFS), and depression (log transformed GDS). For cognitive status, a significant regression was found *F*(*1*,*78*)) = 5.056, *p* = 0.027 the *R*^2^ was.061, indicating that nutritional status explained approximately 6% of the variance in cognitive status. The regression equation is as follows: cognitive status = 3.061 + 0.17. That is, for each one-point increase in nutritional status, the predicted cognitive status increased by approximately 0.17 points. Confidence intervals indicated that we can be 95% certain that the slope to predict cognitive status from nutritional status is between 0.02 and 0.32. For frailty (EFS), a significant regression was found *F*(*1*,*78*)) = 11.764, *p* = 0.001 the *R*^2^ was 0.131, indicating that nutritional status explained approximately 13% of the variance in frailty. The regression equation is as follows: frailty = 13.545 − 0.38. That is, for each one-point increase in nutritional status, the predicted frailty near by approximately 0.38 points. Confidence intervals indicated that we can be 95% certain that the slope to predict cognitive status from nutritional status is between 0.06 and 0.16. No significant regression was found for depression *F*(*1*,*78*)) = 2.963, *p* = 0.089.

### 3.5. Marital Status

Mann–Whitney U test results indicated that, compared to unmarried participants, married participants had significantly lower frailty scores (*z* = −2.396, *p* = 0.017) and depression levels (GDS) (*z* = −2.203, *p* = 0.028), and significantly higher grip strength (*z* = 3.590, *p* < 0.001)

### 3.6. Education

Mann–Whitney U tests were performed to evaluate whether nutritional status, level of depression, cognitive status, and/or frailty differed by participants level of education (high school level vs. graduate level). The results indicated that, in our population, there was no significant difference between participants educated at a high school level and those educated at a graduate level in any of the investigated variables.

## 4. Discussion

The present study aimed to explore the interrelationships between grip strength, cognitive function, nutritional status, depression, and frailty in a cohort of 80 older adults. The findings confirmed our research hypotheses and aligned with the existing literature, highlighting the critical connections between these variables and the importance of integrated strategies to improve the quality of life in older populations [23]. Furthermore, this study brings innovation by not only confirming these correlations but also testing a multidimensional and rapid assessment tool designed to evaluate frailty or pre-frailty stages. This approach facilitates the implementation of preventive strategies, ultimately easing the burden on healthcare professionals while promoting early intervention.

**Hypothesis 1.**  *Correlation between grip strength and cognitive function.*

Our results demonstrated a significant correlation between grip strength and cognitive function. Higher grip strength was associated with better cognitive performance, emphasizing the importance of maintaining muscle strength to help prevent cognitive decline in older adults. This supports prior research showing that grip strength is a reliable indicator of musculoskeletal health and cognitive resilience [24,25,26].

Notably, San Lee et al. (2022) found that weaker handgrip strength was associated with a higher risk of cognitive impairment over time, especially in women [27]. Similarly, Jiang et al. (2022) demonstrated that stronger grip strength correlates with greater brain grey matter volume, supporting cognitive function and potentially protecting against cognitive decline [28]. These studies underscore the value of grip strength as a predictor of cognitive health, particularly in the aging population.

Our study adds to this body of research by focusing specifically on how grip strength correlates with cognitive function and depression in a cross-sectional, multidimensional geriatric assessment [29,30,31,32,33,34].

While prior studies emphasized long-term changes, our findings highlight the potential of grip strength as a practical, real-time marker for assessing cognitive function in older adults, facilitating early intervention in clinical settings. Additionally, our study aligns with previous research by reinforcing the association between physical frailty and cognitive decline [13,35], emphasizing the role of grip strength in predicting frailty-related outcomes such as mortality, disability, and recovery time [36].

Additionally, a novelty of our study is the use of the Squegg™ digital dynamometer to measure handgrip strength in relation to cognitive function, an instrument validated by research in the field [37,38].

**Hypothesis 2.**  *Nutritional status as a predictor of frailty.*

Nutritional status, assessed using the Mini Nutritional Assessment (MNA), emerged as a key predictor of frailty. Adequate nutrition was linked to lower levels of physical and cognitive frailty, consistent with studies showing that nutritional deficiencies exacerbate functional decline and cognitive impairment [39,40]. Previous research, such as that by da Silva et al. [41,42] and Bauer et al. (2013) [43], has demonstrated how nutrient deficiencies accelerate muscle loss and frailty, contributing to a cycle of deteriorating health [44,45].

Our study aligns with these findings but adds value by incorporating a multidimensional assessment that examines how nutrition interacts with other factors such as grip strength and depression. By integrating these variables into a rapid assessment tool l, our study provides a practical tool for healthcare professionals to identify at-risk individuals and implement preventive interventions in a timely manner.

**Hypothesis 3.**  *Impact of marital status on frailty.*

Our findings also confirmed that marital status influences frailty outcomes. Unmarried individuals exhibited higher frailty scores compared to their married counterparts, reflecting the protective role of social support, as highlighted by Kojima et al. (2020) [46] and Chen et al. (2024) [47].

While other studies have noted the association between marital status and cognitive function, our research contributes by showing how this factor influences overall physical frailty in older adults, underscoring the importance of addressing both the social and physical determinants of health [48].

**Hypothesis 4.**  *The effectiveness of the easy to use and multidimensional geriatric assessment tool.*

In integrating data obtained from the Squegg™ digital dynamometer with the four standardized geriatric scales, we developed a valid and reliable assessment tool that aligns with the existing literature on cognitive, functional, nutritional, and psycho-emotional status, yet is more time-efficient and cost-effective. In contrast, other studies have employed a greater number of scales, longer-duration assessments, or traditional dynamometers, which extended the evaluation time. Instead of the MMSE, we used the Rapid Cognitive Test (RCT) [49], which reduces cognitive assessment time by half; for depression, we opted for the 15-item GDS rather than the 30-item version; and, in place of the conventional Jamar dynamometer, we utilized the Squegg™ digital dynamometer, a more ergonomic device that also enables digital data storage.

While the CGA typically takes between 1 and 2 h [50,51] and often requires a multidisciplinary team for data collection, our proposed instrument can be completed in a shorter time and allows for data to be collected by a single healthcare provider. The originality of the study and its value lies in achieving a multidimensional assessment within approximately 10 min, providing a solid foundation for evaluating patients over 65 and for structuring a more tailored treatment plan based on their specific needs.

### 4.1. Clinical Implications

The findings of this study provide crucial insights into the relationships between grip strength, frailty, cognition, depression, and nutritional status in older adults, which can significantly refine geriatric rehabilitation practices. Identifying these interrelated factors enables the development of personalized treatment plans that address the root causes of functional decline in the elderly. Implementing regular grip strength screening in clinical assessments serves as an effective tool to detect early signs of frailty and functional deterioration, allowing for timely, targeted interventions [52,53]. This is particularly important because frailty often leads to a cycle of escalating health issues, which not only diminishes the quality of life for the individual but also places a considerable burden on healthcare systems through increased hospitalizations and long-term care needs [5,54,55,56].

From an economic perspective, the prevention of frailty is paramount for ensuring the sustainability of healthcare systems. Early intervention and management can significantly reduce the need for costly specialized care and long-term support. By addressing frailty at its earliest stages, healthcare systems can avert the high costs associated with treating advanced frailty, which often includes frequent medical consultations, extended hospital stays, and rehabilitation services. Preventive strategies, such as grip strength assessments, offer a cost-effective approach by reducing the incidence of severe disability and dependence, thus lightening the economic strain on public health infrastructures.

Furthermore, focusing on frailty prevention has profound implications for improving the quality of life in older adults by promoting greater autonomy and independence. Frailty increases the risk of disability, which often leads to dependence on family, caregivers, or social services [57].

This can be emotionally and financially taxing for families and communities. In contrast, maintaining muscle strength, proper nutrition, and cognitive health through preventive measures can help preserve functional independence, enabling older adults to remain active and engaged in their daily lives.

Implementing effective prevention programs that encompass physical exercise, proper nutrition, and regular health screenings can greatly reduce the onset and progression of frailty. Evidence from the literature and the current study highlights the importance of multidomain interventions—targeting physical, cognitive, and nutritional factors—that can reduce frailty symptoms and enhance functional capacity and autonomy in older adults [58,59]. Such integrated care approaches [60] not only improve individual well-being but also lessen the broader social and economic burden on communities.

In the long run, a strong focus on frailty prevention is essential not only for improving individual health outcomes but also for promoting social and economic sustainability [61]. Investments in proactive health management and early detection protocols, like the one proposed in this study, can translate into substantial savings for healthcare systems. By supporting the autonomy and well-being of older adults, these interventions contribute to a more sustainable and equitable society, where older populations can continue to thrive with reduced reliance on intensive healthcare services [62].

One of the most significant findings was the strong correlation between hand grip strength (HGS) and several key health factors. Higher grip strength was linked to better cognitive function, improved nutritional status, and lower levels of frailty and depression. These results emphasize the multifaceted role of grip strength as an important marker of both physical and cognitive health. Additionally, the use of the Squegg™ digital dynamometer, which provides accurate and efficient grip strength measurements, enhances the reliability of these assessments and supports its feasibility as a tool for routine clinical practice. This tool has the potential to streamline evaluations and contribute to the earlier detection of health risks in older adults, facilitating timely, personalized interventions.

Our study also revealed the critical role of nutrition in preventing frailty and cognitive decline. Better nutritional status was strongly associated with lower frailty levels and fewer depressive symptoms, underscoring the importance of maintaining adequate nutrition in promoting overall well-being in older adults. These findings suggest that integrating nutritional assessments into geriatric care can significantly enhance the effectiveness of interventions aimed at preventing frailty and cognitive deterioration.

In addition to physical and cognitive factors, the study highlighted the importance of social support, particularly marital status, in influencing health outcomes. Married individuals demonstrated higher grip strength, lower frailty, and lower depression scores compared to their unmarried counterparts, reinforcing the protective role of social connections in maintaining both physical and mental health. This echoes existing research and underscores the need to consider social determinants of health when assessing older adults.

Gender differences were also noted, with men showing significantly higher grip strength compared to women, although no significant differences were observed in nutritional status, depression, frailty, or cognitive function. Among women, stronger grip strength was positively associated with better cognitive function and nutritional status, as well as with being married, while lower grip strength was linked to higher levels of frailty and depression. These findings highlight the nuanced ways in which physical, cognitive, and emotional health intersect in different demographic groups.

Carrying out a comprehensive geriatric assessment is the ideal solution for addressing any pathology of the elderly patient, but the disadvantage is that the known protocols use numerous scales and take a long time. For this reason, identifying a rapid tool that still provides the initial data necessary for an effective treatment, usable in geriatric rehabilitation, but also in other medical specialties, represents a challenge/novelty.

### 4.2. Study Limitations

Although the sample size of 80 participants provides a solid basis for analysis, it may be insufficient to capture the full variability within the elderly population. This limited sample size could restrict the ability to detect certain patterns and associations that might emerge in a larger cohort. Additionally, the cross-sectional design of the study, which measures variables at a single point in time, restricts the ability to establish causality between variables. Thus, it limits conclusions about the relationships among grip strength, cognitive function, nutritional status, and frailty.

The sample’s selection from a single medical facility also constrains the generalizability of the results to elderly populations in other regions or countries, potentially limiting applicability to different demographic or cultural groups. Another limitation concerns the subjective nature of some data, such as responses on self-report questionnaires, which may be influenced by participants’ personal perceptions and memory, introducing potential biases. Furthermore, the categorization of educational attainment was limited to high school and university levels, potentially overlooking distinctions among a broader range of educational backgrounds. Consequently, no significant differences were identified between high school-educated and university-educated participants regarding nutritional status, depression, cognitive status, or frailty.

### 4.3. Future Research Directions

Future studies could address these limitations by employing a larger sample size to confirm and extend the current findings, potentially capturing a broader range of variations within the elderly population. A longitudinal study design would also allow for a more comprehensive understanding of causative relationships among grip strength, cognitive function, nutritional status, and frailty over time.

Including participants from diverse locations and settings could enhance the generalizability of results, supporting findings across different regions and healthcare facilities. Additionally, using objective assessments where possible, rather than relying solely on self-reported data, may reduce the impact of participant bias. Expanding the categorization of educational attainment in future studies would also enable a more nuanced analysis of the relationships between education and health outcomes in the elderly.

In this study, we aimed to identify a viable tool for geriatric rehabilitation; however, our perspective is that this instrument could also be applicable in other medical specialties that assess patients over the age of 65. Future research could explore this tool’s effectiveness and adaptability across various fields, expanding its potential utility as a standardized assessment instrument for older adults in diverse clinical settings.

## 5. Conclusions

The study’s findings, which demonstrate that higher grip strength is associated with improved cognitive function, better nutritional health, and reduced frailty and depression, align closely with patterns observed in the broader literature. In addition, nutritional status was positively associated with cognitive function and inversely related to both frailty and depression, emphasizing the critical role of nutrition in supporting cognitive and emotional health in older adults. Marital status also emerged as an influential factor, with married participants displaying higher grip strength, lower frailty scores, and lower levels of depression compared to unmarried participants.

Notably, this study achieved these insights using a rapid protocol that integrates only four assessment scales and a reliable, user-friendly digital dynamometer, thereby offering an efficient and valid approach for evaluating key health indicators in older adults.

This streamlined assessment protocol has distinct advantages. Its simplicity and speed make it accessible across various medical specialties involved in the care of patients aged 65 and older, enabling a broad range of healthcare providers to effectively identify individuals at risk of frailty and cognitive decline. The inclusion of a digital dynamometer not only provides a practical measure of grip strength—a valuable indicator of overall health—but also enhances the instrument’s feasibility in busy clinical settings due to the device’s ease of use and consistent reliability.

Given its efficiency and validity, this instrument has the potential to serve as a valuable tool for holistic patient assessment, supporting healthcare professionals in delivering targeted, personalized care, as an alternative to the CGA, when time or infrastructure constraints are not met.

## Figures and Tables

**Table 1:** Demographic data.

Item	Variable	N	%
Gender	Male	49	61.3
	Female	31	38.8
Age (Yrs)	65–69	25	31.3
	70–75	36	45.0
	76–80+	19	23.8
Level of education	High education—high school	50	62.5
	Higher education—university studies	30	37.5
Marital Status	Married	58	72.5
	Not married	22	27.5
History of heart disease	No heart disease	36	45.0
	Arterial fibrillation (AFib)	2	2.5
	High blood pressure	42	52.5
History of diabetes mellitus	No	64	80.0
	Type II Diabetes	16	20.0
Dyslipidemia	No	58	73.4
	Yes	22	27.6
Decreased bone density	No	70	87.5
	Osteopenia	2	2.5
	Osteoporosis	8	10.0

**Table 2:** Descriptive statistics for grip strength (HGS), nutritional status (MNA), frailty (EFS), depression (GDS), and cognition (RCT) in scores for men and women.

Gender		N	Mean	Std. Deviation
F	Age	31	73.03	5.37
	Level of education	31	1.29	0.46
	Marital status	31	1.84	0.37
	MNA	31	25.48	2.47
	GDS	31	3.87	2.55
	EDMONTON (EFS)	31	3.45	2.56
	RCT	31	7.19	1.95
	HGS	31	30.82	7.33
M	Age	49	71.67	4.29
	Level of education	49	1.43	0.50
	Marital status	49	1.65	0.48
	MNA	49	24.63	2.38
	GDS	49	3.82	2.78
	EDMONTON (EFS)	49	4.08	2.61
	RCT	49	7.45	1.52
	HGS	49	22.70	6.29

Notes: HGS (grip strength), MNA (nutritional status), EFS (frailty), GDS (depression), RCT (cognition).

**Table 3:** Skewness, kurtosis, and normality tests for MNA, GDS, EFS, RCT, and HGS.

Variable	Skewness			Kurtosis			*p*
	Value	SE	Z	Value	SE	Z	Shapiro–Wilk Test
MNA	−0.536	0.269	−1.99	0.269	0.3	0.896	0.11
GDS	0.847	0.269	3.14	0.145	0.532	0.272	<0.001
EFS	0.459	0.269	1.7	−0.571	0.532	−1.07	0.002
RCT	−0.696	0.269	−2.58	0.427	0.532	0.802	<0.001
HGS	0.228	0.269	0.847	−0.066	0.532	−0.124	0.313

Notes: HGS (grip strength), MNA (nutritional status), EFS (frailty), GDS (depression), RCT (cognition).

## Data Availability

The datasets presented in this article are not readily available because [the data are part of an ongoing study, included in a Ph.D. thesis, that is no public yet].
